# Distinct cortical thickness patterns link disparate cerebral cortex regions to select mobility domains

**DOI:** 10.1038/s41598-021-85058-z

**Published:** 2021-03-23

**Authors:** Inbal Maidan, Anat Mirelman, Jeffrey M. Hausdorff, Yaakov Stern, Christian G. Habeck

**Affiliations:** 1 Laboratory of Early Markers of Neurodegeneration, Center for the Study of Movement, Cognition, and Mobility, Neurological Institute, Tel Aviv Sourasky Medical Center, 6 Weizmann Street, 64239 Tel Aviv, Israel; 2 Department of Neurology, Sackler School of Medicine, Tel Aviv University, Tel Aviv, Israel; 3 Sagol School of Neuroscience, Tel Aviv University, Tel Aviv, Israel; 4 Department of Physical Therapy, Sackler Faculty of Medicine, Tel Aviv University, Tel Aviv, Israel; 5 Department of Orthopaedic Surgery, Rush Alzheimer’s Disease Center, Rush University Medical Center, Chicago, IL USA; 6 Cognitive Neuroscience Division of the Department of Neurology, Taub Institute for Research on Alzheimer’s Disease and the Aging Brain and G.H. Sergievsky Center, Columbia University Irving Medical Center, New York, NY USA

**Keywords:** Motor cortex, Premotor cortex

## Abstract

The cortical control of gait and mobility involves multiple brain regions. Therefore, one could speculate that the association between specific spatial patterns of cortical thickness may be differentially associated with different mobility domains. To test this possibility, 115 healthy participants aged 27–82 (mean 60.5 ± 13.8) underwent a mobility assessment (usual-walk, dual-task walk, Timed Up and Go) and MRI scan. Ten mobility domains of relatively simple (e.g., usual-walking) and complex tasks (i.e., dual task walking, turns, transitions) and cortical thickness of 68 ROIs were extracted. All associations between mobility and cortical thickness were controlled for age and gender. Scaled Subprofile Modelling (SSM), a PCA-regression, identified thickness patterns that were correlated with the individual mobility domains, controlling for multiple comparisons. We found that lower mean global cortical thickness was correlated with worse general mobility (r = − 0.296, *p* = 0.003), as measured by the time to complete the Timed Up and Go test. Three distinct patterns of cortical thickness were associated with three different gait domains during simple, usual-walking: pace, rhythm, and symmetry. In contrast, cortical thickness patterns were not related to the more complex mobility domains. These findings demonstrate that robust and topographically distinct cortical thickness patterns are linked to select mobility domains during relatively simple walking, but not to more complex aspects of mobility. Functional connectivity may play a larger role in the more complex aspects of mobility.

## Introduction

Walking abilities and mobility are critical to safe ambulation and functional independence. Conversely, alterations in gait are associated with and predictive of mortality, morbidity, and multiple adverse health outcomes^1,2^. Furthermore, various groups have generated models of gait that sort discrete gait characteristics into specific gait domains^3–5^. Interestingly, prospective clinical studies have shown that changes in some of these specific gait domains are associated with the development of disparate outcomes (e.g., Alzheimer’s disease, mild cognitive impairment, Parkinsonism, and death)^1,2,6–8^. In addition, these gait domains were used to provide a framework for investigating and interpreting findings related to the neural correlates of gait control^9,10^. Altogether, these findings suggest that the cortical control of gait and mobility and their relationship to specific brain regions and structures is not monolithic. Instead, the relationships between brain structures and walking abilities apparently depend on the specifics of the mobility domain under question.

Neuroimaging studies also support the idea that the links between gait and cortical function are dependent on the specific aspect of gait^10^. Alterations in gait during simple and complex walking tasks and mobility components such as turning and transitions (to and from walking to sitting) have been associated with reduced gray matter volume (GMV) across multiple brain regions^11–16^. Slower gait and reduced step length, two components of the “pace” domain of gait^3^ were associated with reduced mean global GMV^11,14^, reduced frontal^17^, occipital, and hippocampal GMV^11,12,14,15^, lower volumes in cerebellar regions^11,15^, and lower basal ganglia volumes^11,13^. Some studies reported that greater (worse) step-to-step variability of step time, a gait measure related to fall risk, was associated with decreased hippocampal volume^18^ and reduced right parietal lobe^19^. However, these findings were not consistently observed^20^. More generally, whole-brain analyses of the structures that link cortical structures to specific aspects of gait are lacking^13^.

One way of study these relationships is to investigate cortical thickness patterns. Until recently, the methodology of whole brain analysis approaches has been restricted to voxel-based morphometry (VBM), implemented volumetrically across the cortex or as deformation analysis in longitudinal datasets. In VBM, gray matter density and gray matter concentrations are crucial for the interpretation of the results, but voxel density at specific point is meaningless as it is a mixed measure of grey matter including cortical surface area and cortical folding. In contrast, cortical thickness is a selective measure of gray matter that may be more sensitive to cortical atrophy and a loss of gray matter^21,22^ and may be more robust since it does not need to be adjusted for intracranial volume. Therefore, in this study, we use a different whole brain analysis approach that measures cortical thickness across the entire cerebrum. This approach may reveal specific patterns of gray matter loss that correlate to particular gait domains.

The neuroimaging studies that have examined the relationship between gait domains and cortical regions are generally limited in two additional key areas. First, most of the extant literature evaluated gait under relatively simple, single-task walking conditions^13^. That is, the participants were asked to walk at their comfortable speed. In contrast, everyday mobility often takes place as the participant moves around while simultaneously performing one or more tasks^23,24^. In contrast to single-task, “usual-walking”, dual-task walking requires additional cognitive resources, especially those associated with executive function and attention, which involves and calls into play additional brain areas^25–27^. Second, the relationships between GMV and turns and transitions (i.e., moving to and from sitting to walking), putatively two relatively complex tasks, have not been well-studied. While the cortical control that regulates these two important aspects of mobility (i.e., turns and transitions) is apparently different from that of other aspects of gait^28^, little is known about their association with gray matter volumes in specific brain regions.

The present analyses were designed to better understand the relationships between specific domains of gait and mobility and brain structures. As summarized in Table 1, we evaluated 10 domains of mobility, adapting previous classifications^3^ to cover usual-walking, dual-task walking, turns, and transitions. Rather than evaluating GMV, we used measures of cortical thickness since previous work has suggested that it may afford superior measurement properties^29^. As detailed below, we used a novel whole brain approach to study the relationship between cortical thickness patterns and mobility domains. Based on the prospective clinical studies that demonstrated that specific gait domains are related to disparate adverse health outcomes^1,2,6–8^ and based on parallel studies of cortical thickness in cognitive function^29^, we hypothesized that distinct cortical thickness patterns would be selectively associated with specific mobility domains. Furthermore, we hypothesized that these associations would depend on the complexity of the task for example, more frontal regions involved during dual-task walking compared to usual-walking.

**Table 1:** The gait and mobility measures used to assess each task and their grouping into ten mobility domains.

Task	Measures		Domains	Z-score
Usual walk	Stride time (m/s)	1.12 ± 0.01	Rhythm usual	0.04 ± 0.09
	Gait speed (m/s)	0.97 ± 0.01	Pace usual	− 0.29 ± 0.07
	Stride length (m)	1.20 ± 0.02		
	Stride regularity	0.76 ± 0.01	Variability usual	− 0.01 ± 0.03
	Stride time variability	1.54 ± 0.06		
	Step regularity	0.63 ± 0.01	Symmetry usual	− 0.20 ± 0.08
	Step symmetry	0.81 ± 0.02		
Dual-task (DT) walk	Stride time (m/s)	1.23 ± 0.01	Rhythm DT	0.04 ± 0.10
	Gait speed (m/s)	0.84 ± 0.01	Pace DT	− 0.24 ± 0.07
	Stride length (m)	1.15 ± 0.01		
	Stride regularity	0.61 ± 0.01	Variability DT	− 0.03 ± 0.04
	Stride time variability (%)	2.67 ± 0.21		
	Step regularity	0.55 ± 0.01	Symmetry DT	− 0.13 ± 0.07
	Step symmetry	0.92 ± 0.03		
Transitions	Ant-post range (g)	1.94 ± 0.25	Transition	− 0.02 ± 0.03
	Ant-post jerk (g/s)	1.39 ± 0.62		
	Pitch (deg/s)	− 79.58 ± 4.04		
	Roll (deg/s)	50.63 ± 2.85		
Turns	Yaw amplitude (deg/s)	168.96 ± 4.30	Turns	0.03 ± 0.05
	Yaw duration (s)	1.72 ± 0.06		

## Methods

We followed all related tenets of the Declaration of Helsinki and all procedures in this work were approved by the local Columbia University Medical Center Institutional Review Board. All included participants gave their informed written consent.

### Participants

The present analysis is based on a larger cohort study of the whole adult lifespan sample, evaluating the relationship of different cognitive domains with fMRI and various structural features^25^. In this study community-living, healthy participants were recruited using established market mailing procedures to equate the recruitment procedures of young and old adults^30^. Participants who responded were screened over the telephone to ensure that they met basic inclusion criteria (e.g., right-handed, English speaking, no psychiatric or neurological disorders that can affect cognition, normal or corrected-to-normal vision, and not taking any CNS-targeting medications). At initial recruitment, general exclusion criteria included MRI contraindications, hearing impairment, and objective cognitive or functional impairment. All subjects were required to be right handed native English speakers. Exclusion criteria included uncontrolled high blood pressure, current or recent non-skin neoplastic disease or melanoma (but not prostatic carcinoma), active hepatic disease or primary renal disease requiring dialysis, primary untreated endocrine diseases (Well-treated hypothyroidism was not excluded), HIV infection or other medical disorders judged by neurologist to interfere with study, pregnant or lactating (participation allowed 3 months after ceasing lactation), and medications that target the CNS taken within the last month. Subjects with psychiatric issues were excluded if they had a history of psychosis, ECT, current or recent major depressive disorder, bipolar disorder or anxiety disorder. Neurological exclusions included brain disorder such as stroke, tumor, infection, epilepsy, multiple sclerosis, degenerative diseases, head injury (LOC > 5 min), mental retardation, imaged cortical stroke or large subcortical lacunae or infarct or space-occupying lesion (≥ 2 cubic cm), and diagnosed learning disability, dyslexia, or ADHD^25^. Eligible individuals were further screened in person and a Mattis Dementia Rating Scale score of at least 130 was required for inclusion in the study^31^.

### Gait and mobility measures

A small, lightweight sensor that includes a three-axis accelerometer and a three-axis gyroscope (DynaPort; McRoberts, The Hague, the Netherlands) was used for assessment of mobility. The device was worn on the lower back of the participants to assess gait and Timed Up and Go (TUG) test^32,33^. The protocol included walking along a 20-m-long corridor for one minute under two conditions: (1) preferred, usual-walking speed, and (2) dual-tasking; reciting words that start with the letter “A” (cognitive task) while walking. The dual-task walking condition was considered a more complex task that requires motor and cognitive brain resources. The spatio-temporal gait measures obtained from mean values of multiple steps included stride time, gait speed, and stride length. Dynamic features of gait that reflect within subject changes over time during the walk included stride time variability, stride regularity, step regularity, and step symmetry, as previously described^34,35^. These seven gait measures were grouped into four domains based on previous studies^3–5,10^: pace encompassing stride length and gait speed, rhythm including stride time, variability including stride regularity and stride time variability, and symmetry encompassing step regularity and step symmetry. This was performed separately for straight-line, single-task, usual-walking and for straight-line dual-task walking, altogether generating 8 domains (Table 1).

The TUG test consisted of standing up from a chair, walking 3 m at a normal pace, turning around, walking back, and sitting back down in the chair. Acceleration signals were derived from three axes: vertical, mediolateral, and anterior–posterior. Angular velocities were derived from the gyroscope as yaw (rotation around the vertical axis), pitch (rotation around the mediolateral axis), and roll (rotation around the anterior–posterior axis) to generate quantitative measures for three subtasks: sit-to-stand and stand-to-sit transitions and turning^36^. The transition domain (9th domain) included four measures: anterior–posterior range, anterior–posterior jerk, pitch, and roll, while the turn domain (10th domain) included the yaw amplitude and yaw duration^5^. These measures were calculated only from the TUG tests and added two more mobility domains. Before creating these 10 domains (pace simple, single-task/dual-task, rhythm simple, single-task/dual-task, variability simple single-task/ dual-task, symmetry simple/dual-task, transitions, turns), all the mobility measures were normalized by subtracting their means and by then dividing by their standard deviations. After normalizing each of the measures, we averaged the normalized measures within each domain. For example, the pace domain score was calculated by averaging the normalized gait speed and the normalized stride length, giving each of the components similar weight. We hypothesized that each of these gait domains that represents a combined variables reflect independent neuroanatomical substrates that would be quantified using different patterns of cortical thickness.

### MRI acquisition and cortical thickness measures

MRI images were acquired in a 3.0 T Philips Achieva Magnet using a standard quadrature head coil. A T1-weighted scout image was acquired to determine the participant’s position. T1-weighted images of the whole brain were acquired for each participant with an MPRAGE sequence with 180 contiguous 1 mm thick axial slices using the following parameters: TR 6.5 ms, TE 3 ms; flip angle 8°, acquisition matrix 256 × 256, and 256 mm field of view^37^. A neuroradiologist reviewed anatomical scans, and any with potentially clinically significant findings, such as abnormal neural structure, was entirely removed from the sample prior to the current analysis. Cortical thickness was measured using the FreeSurfer analysis package (Version 5.1, https://surfer.nmr.mgh.harvard.edu/fswiki/CorticalParcellation) and resulted in 68 regions of interest (ROIs) and mean global cortical thickness of the entire brain (not just of the 68 ROIs) for all 115 participants^37,38^.

### Statistical analyses

#### Demographics and mobility measures

All variables were evaluated for normality and homogeneity using box plots and scatter plots. Demographic and general mobility measures are reported as mean ± standard deviation for continuous variables and percent for categorical variables. Pearson correlations (not adjusted for age and gender) were performed to examine associations between demographic variables, gait speed and TUG duration as proxies for general mobility and mean global cortical thickness.

#### Subprofile scaling modeling of adjusted thickness mobility relationships

Since cortical thickness very often shows strong associations with age and gender^39^, we residualized both the mobility outcome and the cortical thickness array with respect to age, gender, and their interaction, before performing Subprofile Scaling Modeling (SSM), i.e. a form of PCA (Principal Component Analysis) regression, on the residual data. SSM’s starting point is the following decomposition which would generically hold for any multivariate technique:

$${\mathbf{Y}} = {\mathbf{V}}\;{\mathbf{W^{\prime}}}$$

Here, **Y** is the cortical thickness array, with rows indexing ROIs and columns indexing participants. This matrix was normalized such that both within-row sums across columns and within-column sums across rows yield zero, i.e. the whole brain mean of each participant and the mean image across participants were removed from the data array. **V** is a matrix of principal components with rows indexing ROIs. **W** is a matrix of component scores with the rows indexing participants. Both **V** and **W** have N − 1 columns, where N is the number of participants or observations. (Our normalization of **Y** removed one degree of freedom.) Before performing the SSM analysis, we included an extra step and removed the influence of several nuisance covariates that included age, gender and age by gender interaction from **Y**.

If we write our array as nuisance covariates as

$${\mathbf{NUIS}} = [{\mathbf{age}}\;{\mathbf{gender}}\;{\mathbf{age}}*{\mathbf{gender}}]$$

where **age** and **gender** are mean-centered column vectors of chronological age and gender (1 = men, 2 = women) with as many rows as participants.

The nuisance covariates can be removed from the pattern scores with linear regression:

$$\begin{array}{*{20}l} {{\text{Estimation}}\;{\text{of}}\;{\text{regression}}\;{\text{weights}}\;\upbeta {:}} \hfill & {{\mathbf{W}}\left( {:,{\text{k}}} \right) = [{\mathbf{1}}\;{\mathbf{NUIS}}] \, {{\varvec{\upbeta}}} + {{\varvec{\upvarepsilon}}}} \hfill \\ {\text{Residualizing:}} \hfill & {{\mathbf{rW}}\left( {:,{\text{k}}} \right) = {\mathbf{W}}\left( {:,{\text{k}}} \right) - [{\mathbf{1}}\;{\mathbf{NUIS}}] \, {{\varvec{\upbeta}}},\;\; {\text{k}} = 1 \ldots {\text{N}} - 1} \hfill \\ \end{array}$$

A residualized data array can be written as

$${\text{r}}{\mathbf{Y}} = {\mathbf{V}}\;{\text{r}}{\mathbf{W^{\prime}}}$$

This array is free of any associations with the nuisance covariates; thus patterns derived from it will yield pattern scores whose correlation with the nuisance covariates is zero by design. We similarly adjusted the mobility outcomes and remove the influence of the nuisance covariates.

The adjusted mobility outcomes and cortical thickness arrays were then submitted to standard SSM analysis. First, a PCA was performed on the cortical thickness volumes, then a brain-behavioral regression determined the best fitting set of PCs to predict the gait/mobility measure. The best-fitting PC-set was determined according to the AIC (Akiake’s Information Criterion) as it provides a more inclusive sets of PCs and do not enforce sparsity implicitly like BIC. If a PC does not contribute sufficiently, the bootstrap procedure would down-weight the influence accordingly. The purpose of using the PCA was to account for correlation in the regional thickness values, rather than performing the analysis on a region-wise level, which would lead to unduly conservative multiple-comparison corrections.

Inferential robustness of the ROI-loadings in the derive patterns was assessed with bootstrap resampling. If we write the whole derivation process as a function:

$${\mathbf{pattern}} = {\text{f}}\left( {{\text{r}}{\mathbf{Mob}},{\text{r}}{\mathbf{Y}}} \right)$$

where r**Mob** refers to the residualized mobility outcome, we can obtain bootstrap samples

$${\mathbf{pattern}}* = {\text{f}}\left( {{\text{r}}{\mathbf{Mob}}*,{\text{r}}{\mathbf{Y}}*} \right)$$

where rMob* and rY* refer to resampled data arrays for which participants were sampled with replacement while keeping the assignment of mobility outcome to thickness intact. We generated 10,000 bootstrap samples, enabling us to plot the coverage interval [2.5‰ 97.5‰] of the bootstrap distribution.

There was no apriori sample size calculation since this would require a prior effect size and some Monte-Carlo simulations. However, Type-I error was tightly controlled as too few observations would have degraded the stability of the loadings: one could find a pattern but hardly localized it as the loadings switch signs too much under bootstrap procedure. This was an inherent protection against Type-I errors of commission in identifying significant loadings.

## Results

### Participants

One hundred and thirty participants were included in the current analyses. Participant characteristics are summarized in Table 2 and mobility measures in Table 1. The age of the participants ranged from 27 to 82 years; 64 (55.7%) were women. TUG duration ranged from 6 to 19 s and gait speed ranged from 71 to 140 cm/s. In univariate associations, higher age was associated with lower levels of mean global cortical thickness and slower gait speed, higher (i.e., worse) stride time variability, and longer (i.e., worse) durations of the TUG. Participants with lower values of mean global cortical thickness took longer to complete the TUG and had higher stride time variability. These associations did not persist after adjusting for age and sex. Mean global cortical thickness was not associated with the other gait measures specified in Table 2.

**Table 2:** Participant characteristics and univariate correlations.

				Pearson’s correlation (r)			
	Mean	SD	Min–max	Mean global thickness	Gait speed	Stride time CV	TUG
Age (years)	60.45	13.75	27–82	− 0.462**	− 0.154	0.203*	0.304*
Gender (% female)	55.7						
Mean global thickness (mm)	2.49	0.12	2.16–2.83	–	0.027	− 0.210*	− 0.296*
Gait speed (m/s)	0.97	0.19	0.71–1.40	0.027	–	− 0.259*	− 0.231*
Stride time variability (%)	1.54	0.67	0.46–4.09	− 0.210*	− 0.259*	–	0.215*
TUG duration (s)	11.76	2.31	6–19	− 0.296*	− 0.231*	0.215*	–

*p < 0.05, **p < 0.001.

### Principal component analysis of cortical thickness

**Figure 1:**
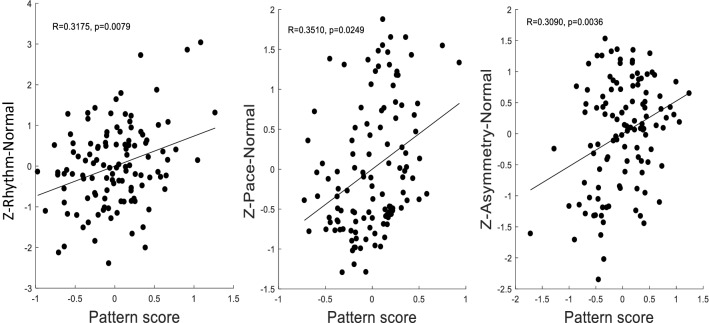
The three patterns which were derived such that their pattern scores correlate maximally with the mobility variables.

Principal components analysis was applied to the thickness measures across all ROIs and 67 principal components (PCs) were derived. We assume invariance across the group and look for differences in degree along one pattern established by a PCA across the whole group. After controlling for age, gender, and age*gender by removing the mean pattern across participants from the data array, three mobility domains were significantly associated with three different subsets of PCs. During single-task, usual-walking, a linear combination of PC1–3 best fit the rhythm domain (R = 0.3175), a linear combination of PC1–6 best fit the pace domain (R = 0.3510), and a linear combination of PC1–2 best fit the gait symmetry domain (R = 0.3090) (Fig. 1). All R-values are bivariate Pearson correlations. No PC combinations were significantly correlated with these domains during dual-task walking or with gait variability, turns or transitions.

### The specific brain regions underlying the PC combinations

**Figure 2:**
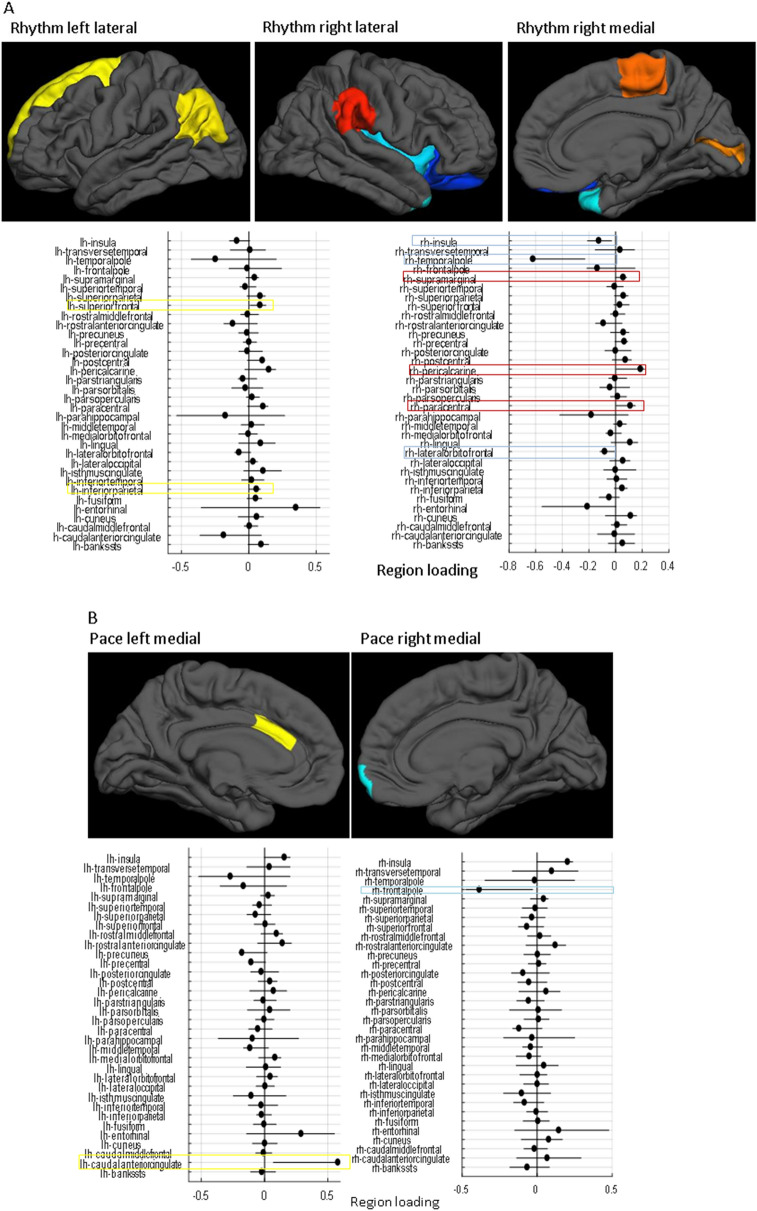

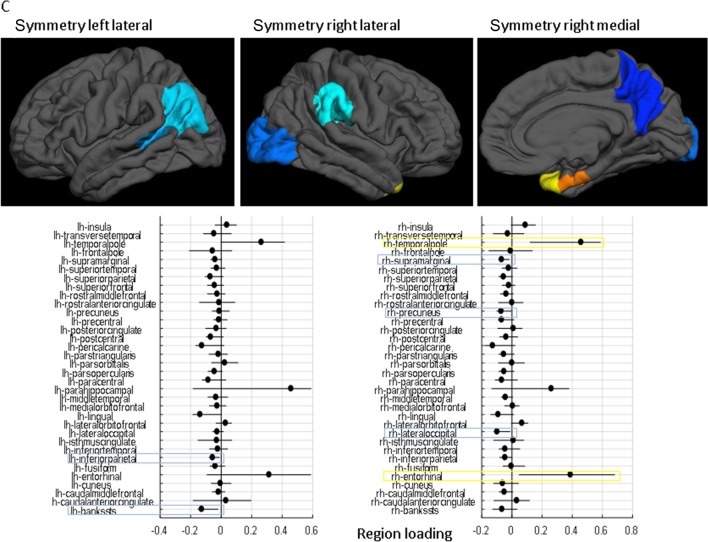
The loading of each brain region in the linear combination of PCs that best fit each of the mobility variables. The top surface plots show regions that were deemed robust in the bootstrap procedure, with hot colors (red shades) indicating positive loadings, and cold colors (blue shades) indicating negative loadings according to the left bars (lh = left hemisphere, rh = right hemisphere). The horizontal line plots show point estimates for the loadings as black dots for each region and indicate the coverage interval [5‰ 95‰] of the 10,000 bootstrap iterations. (**A**) the cortical-thickness pattern constructed from PCs1–3, which is associated with usual-walking gait rhythm, (**B**) the cortical-thickness pattern constructed from PCs 1–6, which is associated with usual-walking gait pace, and (**C**) the specific brain regions constructed from PCs 1–2 which is associated with usual-walking gait symmetry. Positive loadings indicate a relatively thicker regional cortex with the gait measures, while negative loadings indicate a relatively thinner regional cortex.

Gait rhythm during single-task, usual-walking was correlated with higher positive loading in the left inferior-parietal, right pericalcarine, and right paracentral. Negative loadings were observed in the right insula and right temporal pole (Fig. 2A). Pace during singe-task, usual-walking was associated with higher positive loading in the left caudal anterior cingulate and negative loading in the right frontal pole (Fig. 2B). Lastly, gait symmetry during single-task, usual-walking was correlated with higher positive loadings in the right entorhinal, and right temporal pole and negative loadings in the left inferior parietal, left bankssts (i.e., cortical areas around the superior temporal sulcus), right lateral occipital, right inferior parietal, left pars-opercularis, and right supramarginal (Fig. 2C).

## Discussion

In this study, we evaluated the relationship between spatial patterns in cortical thickness and mobility measured in multiple domains. Our novel approach revealed that three distinct patterns of cortical thickness were associated with three different gait domains during simple, single-task usual-walking: pace, rhythm, and symmetry. In contrast, no associations were observed between cortical thickness and more complex mobility measures such as dual-task walking, transitions, or turns.

Several previous studies have shown correlations between changes in brain structural variables and gait impairments^10,14,16,19^. However, our approach of using whole-brain analyses with principal component regression to optimally identify specific brain area patterns associated with different mobility modalities has not been previously applied. The present findings demonstrate selective associations between cortical thickness patterns and mobility domains during relatively simple, single-task walking after controlling for age and gender. Different combinations of six patterns of cortical thickness (PC1–6), each based on 68 ROIs, were correlated to three mobility domains (Supplementary Table 1). Interestingly, specific and different brain regions underlying the PC combinations correlated with rhythm, pace, and symmetry. These correlations included positive and negative associations. The negative associations indicate that better performance is associated with relatively lesser thickness at some ROIs with co-varying greater thickness at other ROIs. In general, this finding of negative and positive associations parallels the select relationships between cognitive domains and cortical thickness patterns that were previously reported^29^. For example, higher positive loadings in the post central area and negative loadings in the parahippocampal area and insula correlated with higher global cognitive function^29^. Together, these findings support the idea that specific cognitive and motor functions are differentially associated with distinct cortical thickness patterns.

The present findings indicate that rhythm was positively correlated with the left inferior parietal lobule, an area that is important for the interpretation of sensory information^40^, right pericalcarine that relates to primary visual areas, and right paracentral that relates to the primary motor cortex. At the same time, rhythm was negatively correlated with the right insula, a region that plays a role in processing bodily sensations^41^, and the right temporal pole, a region that has been linked to semantic memory^42^. In general, these findings indicate that rhythm is associated with greater thickness in sensorimotor brain areas along with lower thickness in ventral areas related to memory and sensation. More generally, these associations indicate that a specific cortical thickness pattern is associated with this domain of gait during usual-walking.

In line with previous studies that examined the correlation between pace and gray matter volume (GMV)^10^, pace (i.e., higher walking speed) was positively correlated with the left caudal-anterior cingulate. This is a brain region that is active during highly demanding tasks that require cognitive control^43^. Pace was also negatively correlated with the right frontal pole region, which acts as a supervisory attentional control system^44^. Interestingly, both regions are part of the higher-order cognitive system and opposite correlations with pace during simple walking may indicate that each play a different role; perhaps the frontalpole is not called into play when the demands of the task are comparatively low as in relatively simple walking. This finding stands somewhat in contrast to other work in older adults and patients with neurodegeneration that has shown the important role of the prefrontal cortex during gait, even during usual-walking^17,26,45,46^. One possible explanation is that for older populations their simple, usual-walking is already challenging, making further demands on higher-order cognitive control. However, if this were the entire explanation, one would expect to see correlations under the dual-task walking condition, which we did not observe. Further work is needed to better understand these findings.

Higher (i.e., better) symmetry was positively correlated with the right temporal pole, a region that is involved in learning and remembering visual-spatial information^47^ and the right entorhinal that play a role in navigation^48^. In contrast, higher symmetry was negatively associated with the left inferior parietal and right supramarginal cortex, regions that are related to body image^49^. Right lateral occipital gyrus and left inferior parietal lobule that are both part of the perception network^50,51^, while the left pars-operuclaris that is important for action observation and imitation^52^. These co-varying thicknesses in brain regions may reflect the complex interaction between different brain networks during gait^10^. Interestingly, symmetry is relatively understudied in the literature in comparison to other measures of gait^10^ and more research needs to be conducted to confirm and elaborate on this interpretation.

Cortical thickness is a volumetric measure that has been considered as a proxy for GMV that does not need to be adjusted for intracranial volume^12^. The advantage of cortical thickness relies on its selectivity to detect changes in gray matter that may be more sensitive to cortical atrophy, compared to other GMV analysis. This can explain the fact that while gait disturbances, mainly from the pace domain, were previously associated with reduced GMV in various brain regions^11,15,16^, our results demonstrate that cortical thickness patterns involve both positive and negative associations with distinct brain regions. While the negative loadings are somewhat counter-intuitive, similar findings were observed in studies of the association between thickness and cognition^29^. In line with studies that used GMV, we found lower thickness in occipital areas^11^, hippocampal areas^53^, and limbic regions^12,15^. However, it is important to emphasize that the specific changes we observed are not an absolute difference in thickness but a relative difference in the context of a covariance pattern. In other words, just observing the thickness of each area separately will not give the same results. Previous GMV studies showed reduced volume in the prefrontal cortex, supplementary motor cortex, and sensorimotor cortex that were associated with worse gait performance^12,15^. In contrast, we observed that greater thickness in the paracentral gyrus was related to a better rhythm of gait (shorter stride time). These contradictory findings may suggest that GMV and cortical thickness are complementary volumetric measures that do not necessarily reflect identical brain properties.

The complexity of the associations between mobility and cortical thickness patterns supports the idea that gait is a multifaceted task that can be viewed as a process that requires "higher-level" cognitive control^45,46,54,55^. Two main locomotor pathways have been identified involving multiple brain areas for the control of gait: the dorsal pathway of cognitive locomotor control and the ventral pathway for emotional locomotor control^56^. The distinct associations that we observed show that the relationships between cortical thickness and gait are not necessarily local and probably involve patterns of correlations and connectivity between different vertices in various spatial dimensions. In addition, different patterns of cortical thickness are apparently linked to specific aspects of gait and the level of task difficulty. Unique patterns were related to pace, rhythm, and symmetry^3^ likely because each represents different gait characteristics^12^ that have distinctions in their cognitive control.

Measures of the performance of more complex aspects of mobility such as dual-task walking, turns, and transitions were not correlated with any patterns of structural cortical thickness. This negative finding is in contrast to our hypothesis that they will be highly correlated with frontal areas associated with cognition. Recent models suggest that the executive locomotor control is activated to supplement automaticity during more complex walking tasks^57,58^. Therefore, with aging and disease, a loss of automaticity increases the reliance on additional cognitive resources^59^. As a consequence, subcortical neural activation is compensated for by more cortical areas^59–61^. As such, it is possible that measures of dual-task walking would correlate with changes in subcortical gray matter that cannot be assessed using methods of cortical thickness. This may explain our negative finding with dual-task walking and the correlation only with usual walking.

In addition, no correlations between cortical thickness patterns and dual task walking may indicate that these facets of mobility are more dependent on functional activity of the brain^26,62^, while structural measures may not be enough to explain their performance. Various functional neuroimaging studies support our observation by showing increased activation and/or connectivity with greater task complexity, irrespective of age and gray matter volume^26,62,63^. The fact that patterns of cortical thickness were not associated with these complex mobility tasks can also be explained in light of the important role of functional brain measures, consistent with the cognitive reserve theory^64^. According to this theory, the performance of a relatively simple task can be within the normal range despite alterations in cortical thickness patterns, as long as sufficient functional activity, considered as a compensatory mechanism, takes place^64^. Alternatively, once task complexity exceeds functional capacity, changes in structural patterns of cortical thickness can become evident and mobility deficits will be observed^65^. Of course, functional connectivity also likely plays a role in the more simple, usual-walking; while cortical thickness patterns were significantly associated with these gait domains, much of the variance was not explained by cortical thickness, suggesting that other factors, like involvement of subcortical areas, connectivity, and perhaps peripheral structure and function, also play an important role.

This study has several limitations. For example, the dual-task included only one type of cognitive task (i.e., reciting words that start with the letter “A”) and measures of dual-task cost were not included in the analysis. Future studies should explore the impact of different cognitive tasks and evaluate the association between dual-task costs and cortical thickness patterns. In addition, in this study, we aimed to filter out the age effects. In the future, it may be of interest to focus on the effects of aging.

In conclusion, applying whole-brain analyses with PCA yielded topographically distinct cortical thickness patterns that were related to selective, relatively simple gait domains. Furthermore, the negative findings with respect to other mobility domains and more challenging conditions underscore the specificity of our findings. The present results motivate the investigation of functional connectivity analyses to evaluate their role in the more complex mobility domains. More generally, these findings emphasize the idea that applying different methodological approaches can reveal unique aspects of the involvement of different cortical regions that can advance our knowledge of how the brain functions to facilitate mobility. In addition, the results support the idea that the cortical control of gait depends on the specific domain.

## Supplementary Information

Supplementary Information.
