# Supplementary material for: Distinct cortical thickness patterns link disparate cerebral cortex regions to select mobility domains
Source: Sci Rep. 2021 Mar 23;11:6600. doi: 10.1038/s41598-021-85058-z (PMC7988162; doi:10.1038/s41598-021-85058-z)
Supplement: Supplementary file 1 — Supplementary Information. [file 41598_2021_85058_MOESM1_ESM.docx]

**Distinct cortical thickness patterns link disparate cerebral cortex regions to select mobility domains**

Inbal Maidan PhD^1,2,3^, [Anat Mirelman](javascript:;),PhD^1,2,3^, Jeffrey M. Hausdorff, PhD.^1,3,4,5^, Yaakov Stern, PhD^6,7^, Christian G. Habeck PhD^6^

^1^Laboratory of Early Markers of Neurodegeneration, Center for the Study of Movement, Cognition, and Mobility, Neurological Institute, Tel Aviv Sourasky Medical Center, Israel

^2^Department of Neurology, Sackler School of Medicine, Tel Aviv University, Israel

^3^Sagol School of Neuroscience, Tel Aviv University, Tel Aviv, Israel;

^41^Laboratory for Early Markers Of Neurodegeneration, Center for the study of Movement, Cognition and Mobility, Neurological Institute, Tel Aviv Sourasky Medical Center, Tel Aviv Israel

^2^Department of Neurology, Sackler Faculty of Medicine and Sagol School of Neuroscience, Tel Aviv University, Tel Aviv, Israel

^4^Department of Physical Therapy, Sackler Faculty of Medicine, Tel Aviv University, Israel

^5^Rush Alzheimer’s Disease Center and Department of Orthopaedic Surgery, Rush University Medical Center, Chicago, Illinois, USA

^6^Cognitive Neuroscience Division of the Taub Institute for Research on Alzheimer’s Disease and the Aging Brain, G.H. Sergievsky Center, Columbia University Medical Center, NewYork, NY

^7^Department of Neurology, Columbia University Medical Center, New York, NY

***Correspondence**:

Dr. Inbal Maidan

Center for the Study of Movement, Cognition, and Mobility, Neurological Institute

Tel Aviv Sourasky Medical Center, 6 Weizmann Street, Tel Aviv 64239, Israel

fax: +972-3-697-4911

e-mail: [inbalm@tlvmc.gov.il](mailto:inbalm@tlvmc.gov.il)

Authors details:

Prof. Anat Mirelman, [anatmi@tlvmc.gov.il](mailto:anatmi@tlvmc.gov.il)

Prof. Jeffrey M. Hausdorff, [jhausdor@tlvmc.gov.il](mailto:jhausdor@tlvmc.gov.il)

Prof. Yaakov Stern, [ys11@cumc.columbia.edu](mailto:ys11@cumc.columbia.edu)

Dr. Christian G. Habeck, [ch629@cumc.columbia.edu](mailto:ch629@cumc.columbia.edu)

**Supplementary Table 1:** The loading of each ROIs in the six PCs

| **Hemisphere** | **Regions** | **PC1** | **PC2** | **PC3** | **PC4** | **PC5** | **PC6** |
| --- | --- | --- | --- | --- | --- | --- | --- |
| Left | bankssts | -0.073 | -0.110 | 0.012 | 0.050 | 0.075 | 0.035 |
|  | caudalanteriorcingulate | -0.084 | 0.137 | -0.209 | -0.193 | -0.477 | 0.420 |
|  | caudalmiddlefrontal | -0.033 | 0.009 | -0.007 | -0.046 | 0.054 | 0.032 |
|  | cuneus | -0.012 | 0.006 | 0.073 | 0.157 | 0.017 | -0.004 |
|  | entorhinal | 0.332 | 0.105 | 0.707 | -0.257 | 0.044 | 0.260 |
|  | fusiform | -0.018 | -0.038 | 0.035 | -0.066 | 0.018 | 0.010 |
|  | inferiorparietal | -0.037 | -0.043 | 0.028 | 0.006 | -0.026 | -0.045 |
|  | inferiortemporal | -0.011 | -0.021 | 0.009 | -0.096 | 0.067 | 0.018 |
|  | isthmuscingulate | -0.048 | 0.008 | 0.116 | -0.155 | -0.035 | -0.160 |
|  | lateraloccipital | -0.019 | -0.018 | 0.020 | 0.002 | -0.065 | -0.038 |
|  | lateralorbitofrontal | 0.006 | 0.036 | -0.069 | -0.036 | 0.031 | 0.085 |
|  | lingual | -0.087 | -0.109 | -0.001 | 0.108 | 0.042 | 0.051 |
|  | medialorbitofrontal | -0.020 | -0.018 | -0.027 | -0.013 | -0.087 | 0.050 |
|  | middletemporal | 0.001 | -0.051 | -0.003 | -0.042 | 0.117 | -0.055 |
|  | parahippocampal | -0.035 | 0.711 | 0.179 | 0.210 | 0.012 | -0.200 |
|  | paracentral | -0.006 | -0.119 | 0.063 | 0.040 | -0.027 | -0.084 |
|  | parsopercularis | -0.072 | 0.012 | -0.002 | -0.058 | 0.035 | 0.025 |
|  | parsorbitalis | 0.026 | 0.007 | -0.013 | -0.155 | 0.139 | 0.152 |
|  | parstriangularis | -0.049 | 0.025 | -0.070 | -0.057 | -0.008 | -0.004 |
|  | pericalcarine | -0.056 | -0.126 | 0.086 | 0.146 | 0.011 | 0.089 |
|  | postcentral | -0.026 | -0.073 | 0.073 | 0.082 | -0.025 | 0.029 |
|  | posteriorcingulate | -0.049 | 0.006 | -0.039 | -0.142 | -0.083 | -0.073 |
|  | precentral | 0.014 | -0.037 | -0.012 | -0.009 | 0.036 | -0.101 |
|  | precuneus | 0.014 | -0.034 | -0.028 | 0.043 | -0.003 | -0.215 |
|  | rostralanteriorcingulate | -0.098 | 0.086 | -0.159 | -0.074 | -0.087 | 0.140 |
|  | rostralmiddlefrontal | -0.063 | 0.030 | -0.030 | -0.096 | -0.033 | 0.104 |
|  | superiorfrontal | -0.042 | -0.019 | 0.074 | -0.093 | 0.000 | 0.005 |
|  | superiorparietal | -0.014 | -0.090 | 0.048 | 0.090 | -0.018 | -0.103 |
|  | superiortemporal | -0.034 | -0.007 | -0.058 | 0.005 | 0.078 | 0.015 |
|  | supramarginal | -0.043 | -0.013 | 0.024 | 0.023 | -0.035 | 0.012 |
|  | frontalpole | -0.097 | 0.022 | -0.057 | -0.350 | -0.208 | -0.316 |
|  | temporalpole | 0.307 | 0.057 | -0.112 | -0.231 | 0.512 | 0.021 |
|  | transversetemporal | -0.050 | -0.015 | -0.026 | 0.209 | 0.039 | 0.073 |
|  | insula | 0.001 | 0.054 | -0.082 | 0.042 | -0.043 | 0.169 |

| **Hemisphere** | **Regions** | **PC1** | **PC2** | **PC3** | **PC4** | **PC5** | **PC6** |
| --- | --- | --- | --- | --- | --- | --- | --- |
| Right | bankssts | -0.030 | -0.064 | 0.013 | 0.063 | 0.160 | 0.031 |
|  | caudalanteriorcingulate | -0.057 | 0.109 | 0.023 | -0.154 | -0.083 | 0.025 |
|  | caudalmiddlefrontal | -0.034 | -0.037 | -0.025 | -0.061 | 0.053 | 0.026 |
|  | cuneus | -0.045 | -0.042 | 0.096 | 0.223 | -0.004 | 0.075 |
|  | entorhinal | 0.671 | -0.151 | 0.020 | 0.123 | -0.493 | -0.192 |
|  | fusiform | 0.053 | -0.063 | -0.069 | 0.054 | -0.039 | -0.009 |
|  | inferiorparietal | -0.047 | -0.016 | 0.028 | 0.005 | -0.019 | -0.020 |
|  | inferiortemporal | 0.018 | -0.086 | -0.030 | 0.064 | 0.012 | -0.086 |
|  | isthmuscingulate | -0.056 | 0.075 | 0.011 | -0.189 | -0.031 | -0.143 |
|  | lateraloccipital | -0.053 | -0.087 | -0.010 | 0.067 | -0.039 | -0.015 |
|  | lateralorbitofrontal | 0.071 | 0.022 | -0.052 | 0.040 | 0.039 | 0.029 |
|  | lingual | -0.063 | -0.066 | 0.066 | 0.184 | 0.051 | 0.081 |
|  | medialorbitofrontal | 0.012 | -0.005 | -0.044 | 0.001 | 0.028 | -0.039 |
|  | middletemporal | -0.021 | -0.041 | 0.007 | -0.034 | 0.050 | -0.014 |
|  | parahippocampal | -0.064 | 0.453 | -0.002 | 0.229 | -0.033 | -0.106 |
|  | paracentral | -0.003 | -0.096 | 0.085 | 0.030 | 0.013 | -0.144 |
|  | parsopercularis | -0.032 | -0.042 | -0.023 | -0.049 | 0.071 | 0.071 |
|  | parsorbitalis | -0.012 | 0.013 | -0.054 | -0.168 | 0.038 | 0.051 |
|  | parstriangularis | -0.035 | -0.041 | -0.050 | -0.041 | 0.052 | -0.020 |
|  | pericalcarine | -0.038 | -0.147 | 0.134 | 0.146 | 0.017 | 0.073 |
|  | postcentral | 0.000 | -0.057 | 0.062 | 0.098 | -0.007 | -0.080 |
|  | posteriorcingulate | -0.038 | 0.055 | 0.011 | -0.165 | -0.021 | -0.125 |
|  | precentral | -0.016 | -0.083 | 0.027 | 0.033 | 0.022 | 0.031 |
|  | precuneus | -0.056 | -0.044 | 0.018 | 0.095 | -0.052 | -0.031 |
|  | rostralanteriorcingulate | -0.030 | 0.033 | -0.116 | -0.011 | -0.095 | 0.101 |
|  | rostralmiddlefrontal | -0.053 | 0.001 | -0.027 | -0.086 | 0.011 | 0.042 |
|  | superiorfrontal | -0.014 | -0.015 | 0.024 | -0.088 | 0.031 | -0.060 |
|  | superiorparietal | -0.020 | -0.059 | 0.030 | 0.048 | -0.015 | -0.053 |
|  | superiortemporal | -0.017 | -0.014 | -0.028 | 0.067 | 0.054 | 0.025 |
|  | supramarginal | -0.053 | -0.043 | 0.019 | 0.020 | -0.021 | 0.043 |
|  | frontalpole | 0.031 | -0.047 | -0.189 | -0.024 | 0.065 | -0.394 |
|  | temporalpole | 0.468 | 0.170 | -0.434 | 0.088 | 0.200 | 0.135 |
|  | transversetemporal | -0.023 | -0.016 | 0.021 | 0.248 | 0.012 | 0.116 |
|  | insula | 0.063 | 0.063 | -0.089 | 0.099 | -0.096 | 0.183 |
